# Late Open Abdominal Aneurysm Reconstruction and Graft Salvage in a Patient With Persistent Endoleak Type II Following Endovascular Aneurysm Repair

**DOI:** 10.7759/cureus.61420

**Published:** 2024-05-31

**Authors:** Nikolaos Giannakopoulos, Afroditi Antoniou, Sofia Tzamtzidou, Dimitra Manou, Theofanis Papas

**Affiliations:** 1 Department of Vascular Surgery, Korgialenio-Benakio Hellenic Red Cross Hospital, Athens, GRC

**Keywords:** type 2 endoleak, evar complications, abdominal aortic aneurysms, open surgical repair, endovascular aortic repair (evar)

## Abstract

This study highlights a case of late open conversion repair (OCR) for persistent Type II endoleak after endovascular aneurysm repair (EVAR), presenting a 78-year-old male with a history of EVAR for an infrarenal abdominal aortic aneurysm. Despite conservative management of the initial endoleak, the aneurysm sac's progressive growth necessitated open reconstruction to salvage the graft. Successful postoperative outcomes emphasize the critical need for meticulous intervention strategies and surveillance in managing persistent Type II endoleaks. This case underlines the importance of a tailored approach, leveraging both endovascular and open surgical techniques, to optimize long-term outcomes and prevent aneurysm rupture in complex cases.

## Introduction

Over the past 30 years, endovascular aortic repair (EVAR) for infrarenal abdominal aortic aneurysms (AAA) has emerged as the preferred treatment option. Additionally, in the last five years, EVAR has largely replaced open aneurysm repair (OAR) due to the latter's association with longer hospital stays and higher rates of 30-day mortality and morbidity [1,2]. However, despite the short-term EVAR advantages, secondary interventions rate up to 15% [3]. Complications such as endoleak type I or persistent endoleak type II leading to aneurysm sac growth are often treated in various centers by open conversion repair (OCR), which ranges from 0.4-22% [1,4,5]. Open conversion repair post-EVAR with EVAR salvage is challenging. On the one hand, the surgeon should solve the problem that could not be solved with endovascular techniques; on the other hand, if possible, the graft should be salvaged [5,6]. This case report aims to highlight the critical need for meticulous intervention strategies and ongoing surveillance in managing persistent type II endoleaks post-EVAR. It demonstrates the successful use of open reconstruction and graft salvage in a complex scenario, emphasizing the importance of a tailored approach to optimize long-term outcomes and prevent aneurysm rupture.

## Case presentation

**Figure 1:**
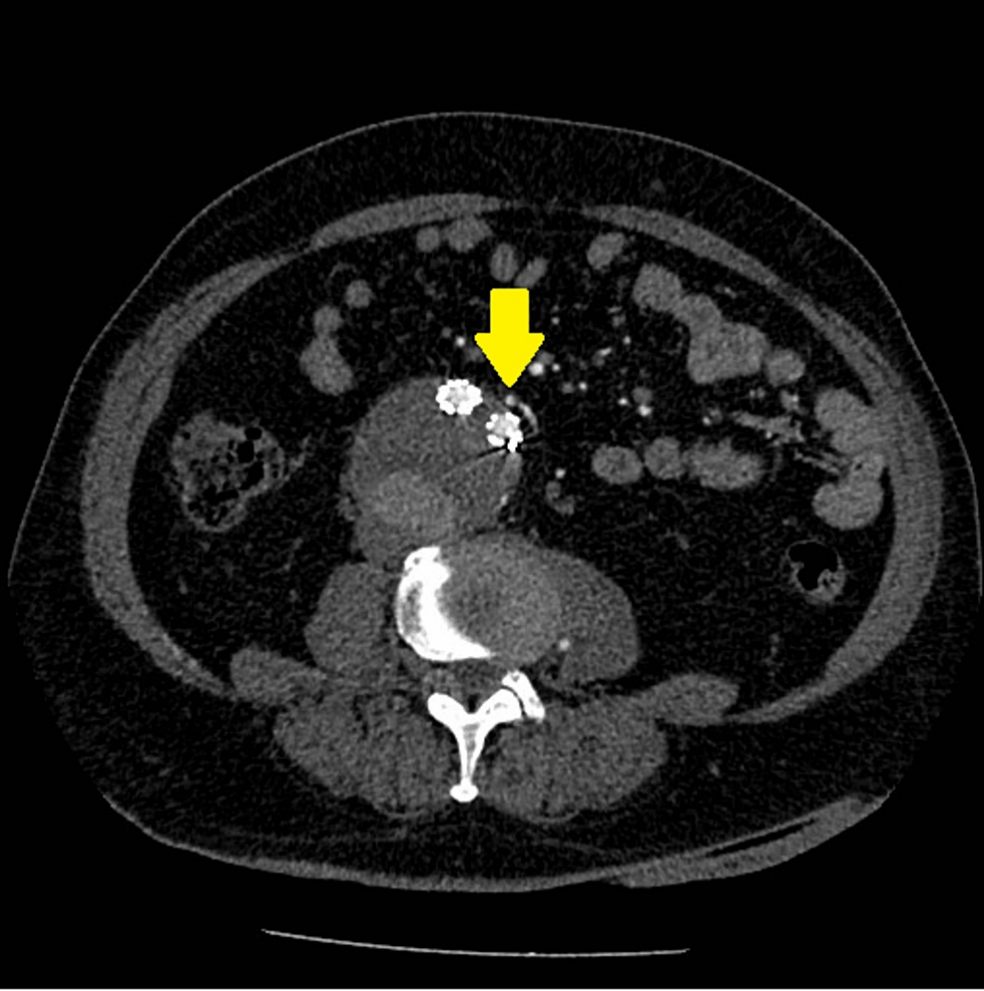
Pre-operative computed tomography angiography shows patent inferior mesenteric artery leading to endoleak type II.

**Figure 2:**
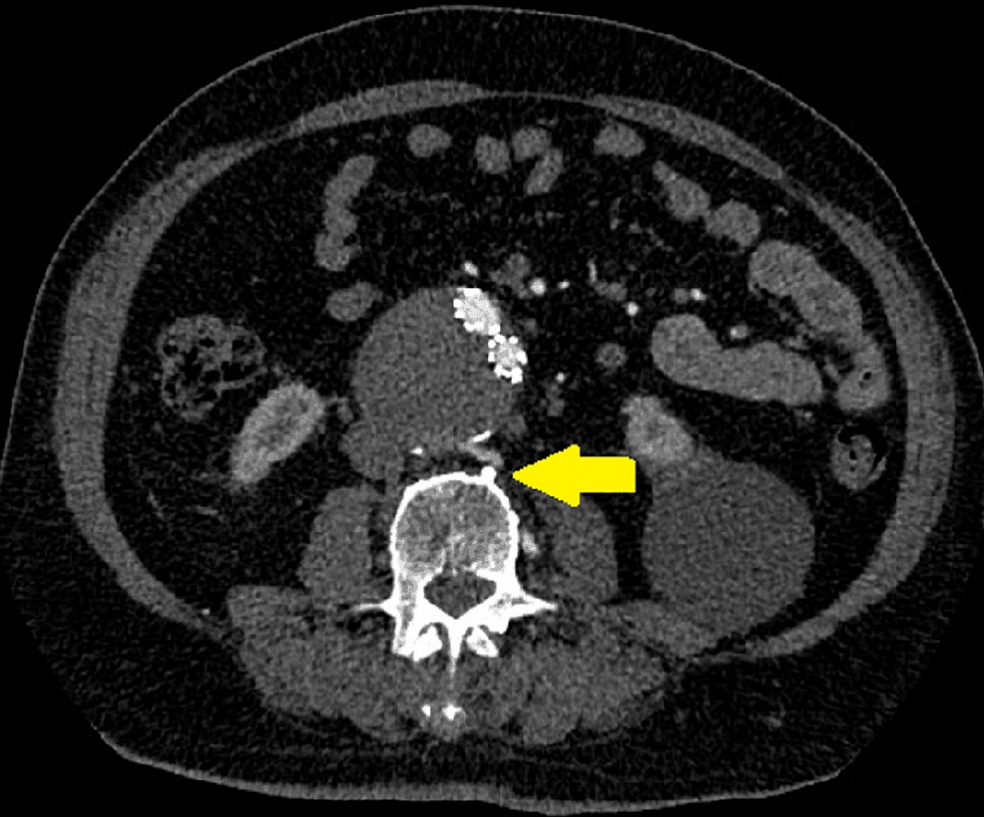
Pre-operative computed tomography angiography shows patent lumbar artery leading to endoleak type II.

This case involves a 78-year-old male who underwent endovascular aneurysm repair (EVAR) for a 5.7 cm infrarenal abdominal aneurysm 14 years ago, in January 2010 at the Vascular Surgery Department of Korgialeneio-Benakeio Hellenic Red Cross Hospital in Athens, Greece. Post-EVAR, he developed a type II endoleak from the patent inferior mesenteric and left lumbar arteries, which was managed conservatively. During his follow-up, he noticed a progressive aneurysm sac growth of roughly 0.2 cm per year. His last follow-up on January 11, 2024, reported a 6 cm aneurysmal sac and still patent inferior mesenteric artery and left lumbar artery. However, the patient was asymptomatic. After repeated consultations for endovascular management of the endoleak, all physicians directed the patient for open reconstruction (Figures 1-2). The patient has a history of hypertension, hyperlipidemia, and a family history of cardiovascular diseases.

**Figure 3:**
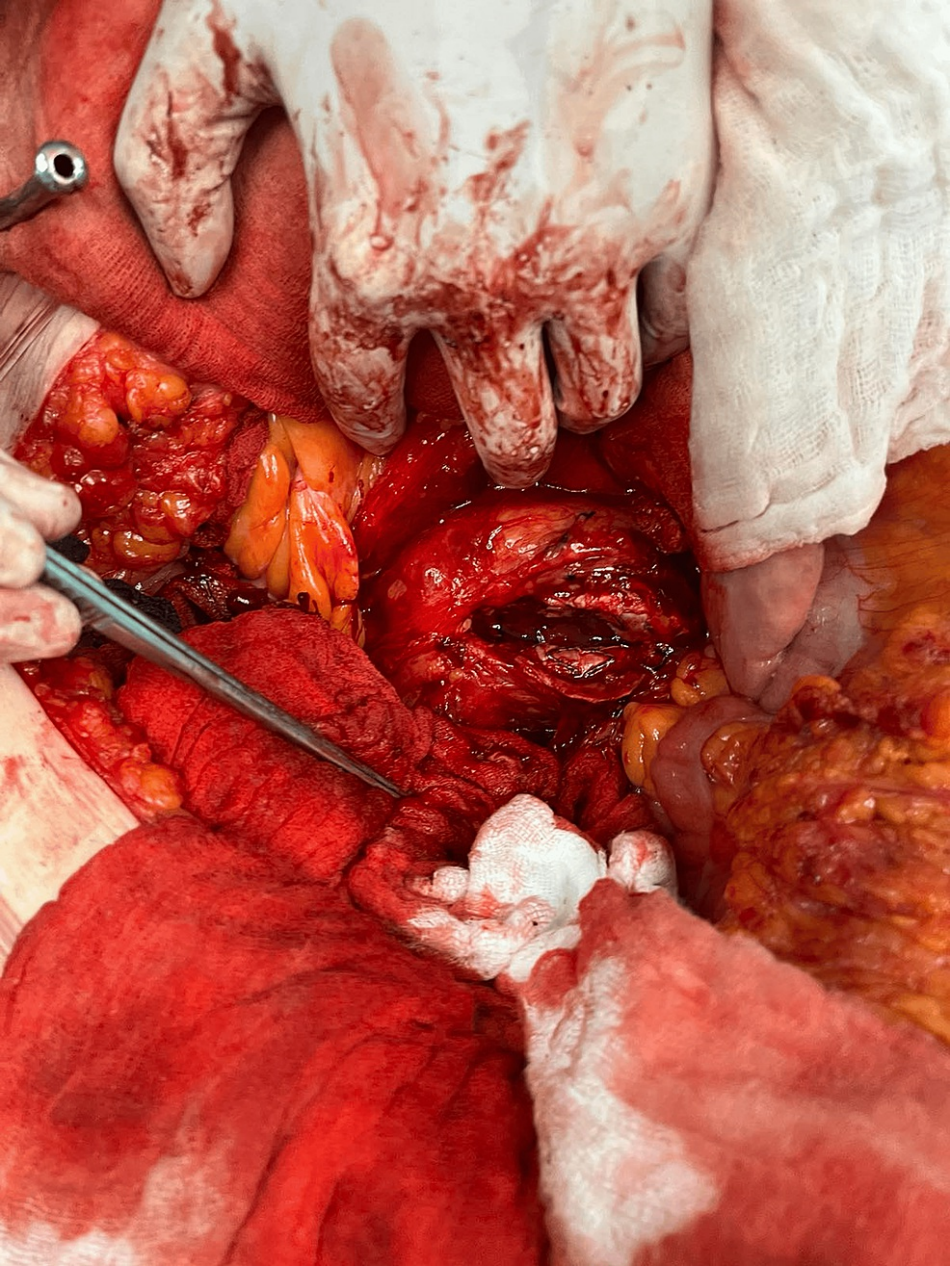
Peri-operative aneurysm sacotomy with transfixed lumbar artery and inferior mesenteric artery shows control of endoleak type II and graft salvage

The patient was planned for elective open reconstruction and EVAR salvage through mid-line laparotomy and by aneurysm sacotomy we managed to ligate the left lumbar artery and transfix the inferior mesenteric artery evacuated aneurysm sac thrombus and finally salvage his graft. The patient didn't experience limb or mesenteric ischemia immediately post-operatively (Figure 3). His post-operative period was uncomplicated and he was discharged on the fourth post-operative day.

**Figure 4:**
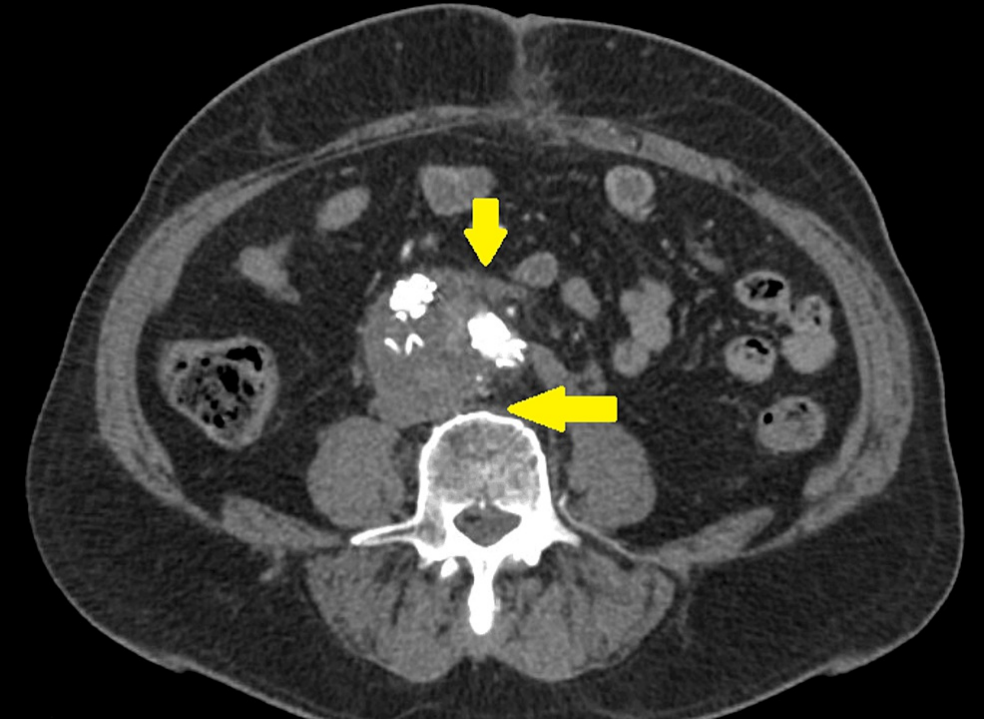
Follow-up computed tomography angiography shows graft salvage and no evidence of endoleak type II.

The patient was planned for follow-up computed tomography on the first post-operative month which showed patent graft and no evidence of endoleak type 2 (Figure 4).

## Discussion

Late open conversion (OCR) after EVAR emerges as a pivotal intervention for managing persistent type II endoleaks, underscored by evolving endovascular techniques and the intricate nature of aortic aneurysm pathophysiology. In this specific patient, the transition from EVAR to OCR was necessitated by the progressive growth of the aneurysm sac and the inability of endovascular techniques to resolve the endoleak. The collective evidence from the studies underscores the nuanced decision-making process and technical expertise required in such transitions, particularly when conventional endovascular solutions are inadequate [3].

A significant revelation from the aggregated data is the identification and management of flow-through type II endoleaks, which present a high risk for aneurysm sac enlargement post-EVAR. These endoleaks, characterized by having both feeding and draining arteries, necessitate meticulous intervention strategies for successful management [7-9]. The case presented by Sano et al. elucidates the successful embolization of an aberrant renal artery contributing to a flow-through type II endoleak, reinforcing the importance of addressing both the inflow and outflow components of the endoleak for effective treatment [8].

The discussions around OCR, as explored in the works by Klonaris et al. and May et al., provide valuable insights into the procedural intricacies and potential complications associated with converting from endoluminal to open repairs. These studies collectively highlight the risks associated with conversion, especially in patients with significant comorbidities, and stress the importance of careful patient selection and preoperative planning to minimize adverse outcomes [5,6].

Moreover, the investigation into persistent type II endoleaks and their impact on long-term EVAR outcomes brings to light the critical need for ongoing surveillance and timely intervention. Persistent endoleaks, especially those involving flow-through mechanisms or emanating from multiple arterial sources, pose a considerable challenge and may necessitate a combination of endovascular and open surgical techniques to achieve definitive sac stabilization and prevent aneurysm rupture [10,11].

## Conclusions

In conclusion, the management of type II endoleaks following EVAR remains a complex and evolving field. Advances in imaging techniques and endovascular tools have improved our ability to diagnose and treat these endoleaks. However, the heterogeneity of aneurysm anatomy and the dynamics of collateral circulation necessitate a tailored approach to each case. The case of our patient, successfully managed through OCR and graft salvage, underscores the need for meticulous intervention strategies and ongoing surveillance. Future research should focus on refining risk stratification models and developing minimally invasive techniques that offer effective solutions while minimizing complications. Collaboration across specialties, including vascular surgery, interventional radiology, and nephrology, is essential for optimizing patient outcomes in the management of type II endoleaks.
